# Hemiarthroplasty and total hip arthroplasty in 30,830 patients with hip fractures: data from the Dutch Arthroplasty Register on revision and risk factors for revision

**DOI:** 10.1080/17453674.2018.1499069

**Published:** 2018-08-06

**Authors:** Sophie Moerman, Nina M C Mathijssen, Wim E Tuinebreijer, Anne J H Vochteloo, Rob G H H Nelissen

**Affiliations:** 1 Department of Orthopedic Surgery, Reinier de Graaf Gasthuis, Delft;;; 2 Department of Surgery/Traumatology, Erasmus MC, Rotterdam;;; 3 Centre for Orthopedic SurgeryOCON, Hengelo;;; 4 Department of Orthopedic Surgery, Leiden University Medical Center, Leiden, the Netherlands

## Abstract

Background and purpose — In the Netherlands about 40% of hip fractures are treated with a hemiarthroplasty (HA) or a total hip arthroplasty (THA). Although these procedures are claimed to have fewer complications than osteosynthesis (i.e., reoperation), complications still occur. Analyses of data from national registries with adequate completeness of revision surgery are important to establish guidelines to diminish the risk for revision. We identified risk factors for revision.

Patients and methods — All patients older than 50 years of age with a hip fracture treated with arthroplasty by orthopedic surgeons and registered in the (national) Dutch arthroplasty register (LROI) were included in the study. In this register, patient characteristics and surgical details were prospectively collected. Revision surgery and reasons for revision were evaluated. A proportional hazard ratio model for revision was created using competing risk analysis (with death as competing risk).

Results — 1-year revision rate of HA was (cumulative incidence function [CIF] 1.6% (95% CI 1.4–1.8) and THA 2.4% (CI 2.0–2.7). Dislocation was the most common reason for revision in both groups (HA 29%, THA 41%). Male sex, age under 80 years, posterolateral approach, and uncemented stem fixation were risk factors for revision in both THA and HA. THA patients with ASA classification III/IV were revised more often, whereas revision in the HA cohort was performed more often in ASA I/II patients.

Interpretation — After arthroplasty of hip fractures, both a posterolateral approach and an uncemented hip stem have higher risks for revision surgery compared with an anterolateral approach and an cemented stem.

Arthroplasty surgery for acute hip fractures is performed in large numbers worldwide. In the Netherlands about 21,000 hip fractures occur annually (NVT and NOV 2016). In about 40% of these cases a hemi- (HA) or total hip arthroplasty (THA) is used (Opendisdata.nl 2017). Although these latter procedures are claimed to have fewer complications than osteosynthesis of the fractured hip, complications still occur (Gao et al. 2012). Analysis of observational data from national registries will more readily give data that can be of clinical value, but such studies are rare (Gillam et al. 2010, Leonardsson et al. 2012b, Gjertsen et al. 2014). A meta-analysis demonstrated a lower risk of reoperation and better function after THA compared with HA (Hopley et al. 2010); a more recent review found comparable outcomes between (bipolar) HA and THA (Wang et al. 2015). None of these studies used national registry data. Also, other issues like the use of a cemented or an uncemented stem, an unipolar or a bipolar HA, and what surgical approach is best to use still remain open (Leonardsson et al. 2012a, Gjertsen et al. 2014, Rogmark and Leonardsson 2016). Therefore, we performed an analysis into failure mechanisms (i.e., end-point revision surgery and reasons for revision) of hemiarthroplasties and total hip arthroplasty using data from the national Dutch Arthroplasty Register (LROI).

## Patients and methods

All acute hip fractures treated with a HA or a THA by orthopedic surgeons that were registered in the LROI between 2007 and 2017 were included in the study. Patient characteristics (sex, age at procedure, ASA classification, smoking, and BMI) and surgical details (approach, type of fixation, and type of implant) are prospectively registered (van Steenbergen et al. 2015). All records in the LROI are linked by the encrypted citizen service number unique to each Dutch inhabitant. All revision operations during which components are replaced as well as reasons for revision are also registered in the database. The citizen number allows these revisions to be linked to the primary procedure. Reason(s) for revision surgery are coded in the database with a multiple response variable set: dislocation, peri-prosthetic fracture, infection, loosening femoral component, loosening acetabular component, cup/liner wear, and other reasons.

For this study we included all registered patients older than 50 years of age, treated with a THA or HA for an acute hip fracture. The LROI has a completeness for primary THA (independent of indication for THA) of 98%, and 88% for revision arthroplasty (van Steenbergen et al. 2015). The completeness of primary HA increased from 70% in 2013 to 88% in 2015 (Landelijke Registratie Orthopedische Implantaten 2013, 2015). In the Netherlands, HA for hip fracture is performed by both orthopedic and trauma surgeons, THA for acute fractures is performed only by orthopedic surgeons. As the registration in LROI by trauma surgeons only started in 2014 and completeness is low, patients treated by trauma surgeons are not included in the current study.

### Statistics

Baseline characteristics for THA and HA are compared with Student’s t-test for continuous variables and the chi-square test for categorical variables. We considered differences between groups to be statistically significant if the p-values were less than 0.05.

The high risk of mortality after arthroplasty surgery is an important competing risk for revision operations. Due to the effect of the competing risk (in this case death) there is a chance of potential under- or overestimation of incidence of reoperations using a Kaplan–Meier analysis (Gillam et al. 2010, Keurentjes et al. 2012, van der Pas et al. 2017). If, for example, an uncemented prostheses in this study was applied to a healthier population with a lower incidence of death, the probability of revision would be higher for that group. For this reason competing risk analysis was performed with STATA 11.2 (StataCorp, College Station, TX, USA) using the Cox model (Ranstam and Robertsson 2017). The estimated cumulative incidence functions (CIF) for revision are presented in graphs for both THA and HA. These CIFs were compared using the Pepe and Mori test for equality of CIF across groups (Pepe and Mori 1993). Revision was defined as the exchange, addition, or removal of one or more components as registered in the LROI. Implant revision rate was calculated at 1 and 5 years postoperatively.

Furthermore, CIFs for revision were made for each covariable separated for HA and THA. Covariables used were sex, age (< 80 years vs. ≥ 80 years) (80 years was chosen since mean age was 80 years, range 50–107 years), ASA classification (I/II vs. III/IV), smoking status (yes/no), normal weight (BMI 18.5–25) was compared with overweight (BMI 25–30), type of approach (posterolateral [53%] or not posterolateral (anterolateral [12%], straight lateral [33%], and anterior [2%]), and type of stem fixation (cemented vs. uncemented). A hybrid THA was classified according to whether the stem was cemented or not, in order to be able to compare with HA. Finally, HA type of head (unipolar vs. bipolar head) was added to the analysis.

The Cox model in a multivariable approach with more covariables produces hazard ratios (HR) with 95% confidence intervals (CI). The estimated coefficients of the variables were tested if they were constant with time and if time interactions were statistically significant. The variables were entered as time-varying covariables in the model when the proportional hazards assumption was violated. Separate proportional hazard models with hazard ratios (HR) are presented for HA and THA.

### Ethics, funding, and potential conflicts of interest

Ethical approval was not required for this study. The Department of Orthopaedic Surgery and the Orthopaedic Research Foundation in Reinier de Graaf Hospital receive grants from Zimmer Biomet. The company (Zimmer Biomet) was not involved in this study. No conflicts of interest to declare.

## Results

30,830 acute hip fractures treated with a HA or a THA were registered in the LROI database between 2007 and 2017. In 22,675 fractures a HA was performed and in 8,155 a THA. 79% received a unipolar HA, 20% a bipolar HA, and 1% a monoblock HA (Table 1).

**Table 1.** Baseline characteristics and surgical details of patients with a hip fracture treated with a total hip arthroplasty (THA) or a hemiarthroplasty (HA)

Factor	THA	HA	Missing
	n = 8,155	n = 22,675	
Sex, female	70% (5,672/8,141)	70% (15,938/22,644)	45
Age, mean (SD)	71 (9.2)	83 (7.7) ^b^	12
ASA, I/II	74% (5,710/7,743)	40% (8,855/22,001) ^b^	1,085
Smoking**^a^**	17% (526/3,170)	8% (729/8,764) ^b^	18,896
BMI, mean (SD)**^a^**	25 (7.3)	24 (9.4) ^b^	17,062
Posterolateral approach	60% (4,790/8,046)	53% (11,860/22,462) ^b^	322
Uncemented stem fixation	57% (4,584/8,036)	34% (7,578/22,442) ^b^	352
Unipolar HA		79% (17,123/21,685)	990

a Smoking and BMI have been registered in the LROI database since 2014.

**^b^**P < 0.001.

### Revision rate

**Figure 1.**
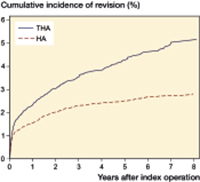
Cumulative incidence function (CIF) of revision estimates from competing risks data (1 – survival) for patients treated with HA and THA (n = 30,830).

1-year revision rate in HA was (CIF [95% CI]) 1.6% (1.4–1.8) and 5-year 2.5% (2.3–2.8). 1-year revision rate in THA was 2.4% (2.0–2.7) and 5-year 4.3% (3.8–4.8) (Figure 1, Table 2, see Supplementary data). Revision rate was higher in THA (p < 0.001).

**Table 2.** Cumulative incidence function (CIF) estimates from competing risks data (1-survival) for patients treated with HA and THA

Factor	Cumulative incidence of revision	
	after 1 year	after 5 years
HA	1.6% (1.4–1.8%)	2.5% (2.3–2.8%)
THA	2.4% (2.0–2.7%)	4.3% (3.8–4.8%)

### Reasons for revision

In 435 HA patients 1 reason for revision was given, in 66 patients multiple reasons were given (153 reasons in 66 patients). Dislocation, periprosthetic fracture, and infection were the most common reasons for revision. In 228 THA patients 1 reason for revision was given, in 70 patients multiple reasons (156 reasons in 70 patients). Dislocation was the most common reason for revision (41%) (Table 3).

**Table 3.** Reasons for revision after hemiarthroplasty (HA) or total hip arthroplasty (THA) for hip fractures

Factor	HA	THA
	n = 501	n = 298
Single reason for revision, n	435	228
Dislocation, n (%)	128 (29)	94 (41)
Peri-prosthetic fracture, n (%)	58 (13)	28 (12)
Infection, n (%)	68 (16)	26 (11)
Loosening of femoral component, n (%)	15 (3)	25 (11)
Loosening of acetabular component or cup/liner wear, n (%)	n/a	18 (8)
Other reasons, n (%)	166 (38)	37 (16)
Multiple of above-mentioned reasons, n	66	70

### Risk factors for revision

Male sex, age below 80 years, ASA classification I/II, a posterolateral approach, and uncemented fixation were risk factors for revision in HA in an univariable analysis risk (Table 4, Figure 2, see Supplementary data). A proportional hazard ratio model using all significant factors showed that male sex, age below 80 years, ASA I/II, a posterolateral approach, and uncemented fixation are risk factors for revision (Table 5). Age and ASA classification were time-varying covariables, meaning that the influence of these variables changes over time. For example, age is no risk factor for revision in the first year after the fracture but becomes one in the years thereafter.

**Table 4.** Factors associated with revision in hip fracture patients after hemiarthroplasty (HA) and total hip arthroplasty (THA) in a univariable analysis with a hazard analysis

Factor	HA		THA	
	HR	95% CI	HR	95% CI
Sex, female (vs. male)	0.78^b^	0.65–0.94	0.61^b^	0.48–0.77
Age, ≥ 80 (vs. < 80 years)	0.55^b^	0.46–0.65	0.44^b^	0.29–0.67
ASA, III–IV (vs. I–II)	0.84	0.70–1.01	1.37^a^	1.06–1.76
Smoking, yes (vs. no)	1.40	0.90–2.18	1.70^a^	1.02–2.83
Weight, obesity (vs. normal BMI)	0.90	0.67–1.22	1.37	0.86–2.17
Approach, non-posterolateral				
(vs. posterolateral)	0.67^b^	0.56–0.80	0.68^a^	0.54–0.88
Stem fixation, cemented				
(vs. uncemented)	0.61^b^	0.51–0.73	0.73^a^	0.57–0.93
Type of HA, bipolar (vs. unipolar)	0.91	0.73–1.14		

HR = hazard ratio.

a P < 0.05

b P < 0.001

**Table 5.** Factors associated with revision in hip fracture treated with a total hip arthroplasty (THA) or a hemiarthroplasty (HA) in a multivariable approach with hazards model with time-varying covariables

Factor	HA		THA	
	HR	95% CI	HR	95% CI
Approach^a^, non-posterolateral				
(vs. posterolateral)	0.67	0.55–0.81	0.70	0.55–0.90
Stem fixation^a^, cemented				
(vs. uncemented)	0.63	0.52–0.75	0.71	0.55–0.91
ASA^b^, III–IV (vs. I–II)	0.72^d^	0.62–0.83	1.46	1.13–1.90
Age^c^, ≥ 80 (vs. < 80 years)	0.59^d^	0.50–0.70	0.52^d^	0.55–0.91
Sex^c^, female (vs. male)	0.80	0.66–0.97	0.65	0.51–0.83

HR = hazard ratio.

a Variables with direct effect on outcome.

b Measured confounder with direct effect on choice of HA or THA.

c Measured confounders with effect on ASA.

d Time-varying covariables,

Confounder with direct effect on revision: HA/THA choice (not accounted for by stratification).

Male sex, age below 80 years, smoking, a posterolateral approach, and uncemented stem fixation were risk factors for revision in THA in an univariable analysis. ASA classification was not a clear risk factor (p = 0.09) (Figure 2, Table 4). A proportional hazard ratio model showed that male sex, younger age, ASA III/IV, a posterolateral approach, and an uncemented stem were associated with more revisions (Table 5). Age was a time-varying covariable meaning that the hazard of age on revision changes over time.

### Specific reason for revision in factors associated with revision

In both THA and HA a fracture as a reason for revision was more common in an uncemented prosthesis (HA 28% vs. 2%, THA 15% vs. 6%) (Table 6, see Supplementary data).

**Table 6.** Reason for revision in factors associated with revision in hip fracture treated with a total hip arthroplasty (THA) or a hemiarthroplasty (HA)

Factor	HA			THA		
	Dislocation	Fracture	infection	Dislocation	Fracture	Infection
All	128/435 (29%)	58/435 (13%)	68/435 (16%)	94/228 (41%)	28/228 (12%)	26/228 (11%)
Sex						
Male	44/142 (31%)	16/142 (11%)	33/142 (23%)	35/94 (37%)	17/94 (18%)	11/94 (12%)
Female	84/293 (29%)	42/293 (14%)	35/293 (12%)^a^	59/134 (44%)	11/134 (8%)^a^	15/134 (11%)
Age						
< 80 years	53/222 (24%)	19/222 (9%)	27/222 (12%)	81/207 (39%)	26/207 (13%)	25/207 (12%)
≥ 80 years	75/213 (35%)^a^	39/213 (18%)^a^	41/213 (19%)	13/21 (62%)	2/21 (10%)	1/21 (5%)
ASA						
I/II	54/209 (26%)	21/209 (10%)	29/209 (14%)	56/139 (40%)	17/139 (12%)	14/139 (10%)
III/IV	73/208 (35%)^a^	34/208 (16%)	38/208 (18%)	32/75 (43%)	10/75 (13%)	12/75 (16%)
Approach						
Non-posterolateral	31/165 (19%)	25/165 (15%)	30/165 (18%)	24/74 (32%)	9/74 (12%)	11/74 (15%)
Posterolateral	96/262 (37%)^a^	32/292 (12%)	38/262 (15%)	70/152 (46%)	18/152 (12%)	15/152 (10%)
Fixation						
Cemented	81/243 (33%)	5/243 (2%)	52/243 (21%)	42/82 (51%)	5/82 (6%)	11/82 (13%)
Uncemented	46/183 (25%)	52/183 (28%)*	16/183 (9%)*	51/142 (36%)^a^	22/142 (15%)^a^	14/142 (10%)

a P ≤ 0.05.

In HA, dislocation as a reason for revision was more common in younger patients (35% vs. 24%), ASA III/IV patients (35% vs. 24%), and a posterolateral approach (37% vs. 19%). A fracture was more common in older HA patients (18% vs. 9%). Infection was more common amongst male patients (23% vs. 12%) and a cemented prosthesis (21% vs. 9%).

In THA dislocation as a reason for revision was more common in a cemented prosthesis (51% vs. 36%). A fracture as a reason for revision was more common in the male sex (THA 18% vs. 8%). 

## Discussion

Revision rate of THA was higher compared with the revision rate of HA. The 5-year revision rate of an HA was 2.5% and 4.3% in THA, which is in contrast to the results from randomized trials, which showed no difference between HA and THA (van den Bekerom et al. 2010, Hedbeck et al. 2011) However, patients included in these randomized trials were less frail than the average hip fracture patients. The HA group in our registry study contained patients with more frailty (higher age, higher ASA classification) than the THA group, therefore the threshold for a surgeon to decide to revise was probably higher in the HA group.

In our study, dislocation was the most common reason for revision in both HA (29%) and THA (41%). Acetabular erosion (prevalence is 2–41%) is a theoretical indication to perform a revision in a painful HA (Baker et al. 2006). In the LROI, acetabular erosion as reason for revision cannot be registered. Patients who were revised for acetabular erosion were classified in the “other” category (38%). How many patients in this category had acetabular erosion is unclear.

Male sex and age below 80 years were risk factors for revision surgery in THA and HA. This in accordance with data from the Norwegian and British register (Stafford et al. 2012, Rogmark et al. 2014). Younger patients are likely to be more demanding regarding hip function after surgery, thus even revision for moderate postoperative complaints is more likely. Males have a higher occurrence of periprosthetic fractures, which may lead to a higher revision rate (Table 6) (Australian Orthopaedic Association National Joint Replacement Registry 2016).

In HA, ASA classification I/II was a risk factor for revision; however, in THA ASA classification III/IV was a risk factor for revision. This contradiction is probably explained by the selection bias of THA and HA. We believe THA patients with an ASA classification of III/IV are less frail than HA with an ASA classification of III/IV, while a surgeon will choose an HA in the frailest patients (i.e., shorter surgical time and less blood loss; Blomfeldt et al. 2007). These frail HA patients (ASA classification III/IV) are unlikely to undergo revision due to higher risks but also to lower demand on functionality of these patients. In THA these ASA classification III/IV patients have a higher risk of revision compared with ASA classification I/II. Comorbidities like diabetes mellitus might cause this higher rate of infection (Dale et al. 2011). A British and Norwegian register study has shown the same tendency for higher revision in higher category ASA patients in THA for hip fracture (Dale et al. 2011, Stafford et al. 2012).

A posterolateral approach was a risk factor for revision in both HA and THA. 2 large register studies showed that the posterolateral approach led to more dislocations (Leonardsson et al. 2012b, Rogmark et al. 2014). However, patient reported outcome measurements (PROMs) used in the registry study in Norway showed that the posterior approach gave less pain, fewer walking problems, and better QoL than the lateral approach (Kristensen et al. 2016). Using a dual mobility cup may reduce dislocation risk when using a posterolateral approach (Batailler et al. 2017, De Martino et al. 2017, Tabori-Jensen et al. 2018).

Uncemented stems were a risk factor for revision in both HA and THA. Peri-prosthetic fractures are more common in uncemented prostheses (both HA and THA), probably as a result of trying to create a press-fit situation in the weaker (osteoporotic) bone (Moerman et al. 2017). This increased risk of periprosthetic fracture in uncemented prostheses must be weighed against the potential complications of cementing such as bone cement implantation syndrome (BCIS) (Donaldson et al. 2009).

Bipolar prostheses are developed to reduce the risk of erosion of the acetabulum. We did not find any difference in revision hazards between unipolar and bipolar heads. 79% of the Dutch hip fracture patients treated with HA receive a unipolar head. Costs for bipolar heads in the Netherlands are about double the costs of unipolar heads. The Swedish register showed more reoperations with bipolar heads (Leonardsson et al. 2012b) and the Australian register found lower reoperation rates with bipolar heads (Gillam et al. 2010). Reasons for these conflicting data may be the difference in hemiarthroplasty populations in Australia, Sweden and the Netherlands.

The NICE guideline (NICE 2011) for hip fractures advises against use of monoblock prostheses. In our register only 164 (0.8%) of all HA were monoblock prostheses. Therefore no analysis on these monoblock prostheses was performed.

### Strengths and limitations

This is the first nationwide Dutch study on HA and THA in acute hip fractures using data from the Dutch Arthroplasty Register (LROI). Previously the Scandinavian, British, and Australian registers have published their results (Gillam et al. 2010, Leonardsson et al. 2012b, Stafford et al. 2012, Gjertsen et al. 2014). The added value of these Dutch results is important, since each country has its own specific health-care organization. As for the Netherlands, a quality mark for hip fractures was that surgery has to be performed within 24 hours of admittance which may cause differences in outcome between registers. Furthermore, this study includes both HA and THA data for acute hip fractures. Observational data studies for THA in hip fractures are sparse, thus knowledge on this subject has to be extended, since the proportion of hip fracture patients treated with THA is increasing. The proportion hazards model clearly assigns risk factors for revision, which is of clinical importance and may guide treatment of these often frail patients in order to minimize the perioperative risks.

A limitation of the study is the incomplete registration of HA for acute hip fractures (but still 88% completeness). Follow-up of hip fracture patients is limited because of the high mortality rate (1-year mortality is around 20%). There are a limited number of patient characteristics registered in our national registry. Alcohol use, for instance, was not registered although it influences revision rate (Johnston and Parker 2014, Kosola et al. 2017). Because of this limited number of patient characteristics, there is potential for residual confounding. Furthermore, only revision operations in which components are replaced are registered in the database. Reoperations without component (re-)placement (like debridement of the wound and the prosthesis without liner exchange in the case of acute infection) are not registered in the LROI database.

In summary, revision rates in both HA and THA after an acute hip fracture are considerable. Avoidance of both an uncemented stem and a posterolateral approach may reduce the revision rate.

### Supplementary data

Tables 2 and 6, and Figure 2 are available as supplementary data in the online version of this article, http://dx.doi.org/ 10.1080/17453674.2018.1499069

Design of study: SM, NM, RN, AV. SM and WT did the statistical analysis. SM, NM, RN, and AV wrote the manuscript. All authors read and approved the final manuscript.

*Acta* thanks Torben B Hansen and other anonymous reviewers for help with peer review of this study.

## Supplementary Material

Supplemental Material
