# Supplementary material for: Hemiarthroplasty and total hip arthroplasty in 30,830 patients with hip fractures: data from the Dutch Arthroplasty Register on revision and risk factors for revision
Source: Acta Orthop. 2018 Aug 6;89(5):509–14. doi: 10.1080/17453674.2018.1499069 (PMC6202728; doi:10.1080/17453674.2018.1499069)
Supplement: Supplemental Material [file IORT_A_1499069_SM1333.pdf]

Supplementary data

Table 2. Cumulative incidence function (CIF) estimates from competing risks data (1-survival) for patients treated with HA and THA

Factor	Cumulative incidence of revision after 1 year	after 5 years
HA	1.6% (1.4–1.8%)	2.5% (2.3–2.8%)
THA	2.4% (2.0–2.7%)	4.3% (3.8–4.8%)

Table 6. Reason for revision in factors associated with revision in hip fracture treated with a total hip arthroplasty (THA) or a hemiarthroplasty (HA)

	HA			THA		
Factor	Dislocation	Fracture	infection	Dislocation	Fracture	Infection
All	128/435 (29%)	58/435 (13%)	68/435 (16%)	94/228 (41%)	28/228 (12%)	26/228 (11%)
Sex						
Male	44/142 (31%)	16/142 (11%)	33/142 (23%)	35/94 (37%)	17/94 (18%)	11/94 (12%)
Female	84/293 (29%)	42/293 (14%)	35/293 (12%) [a]	59/134 (44%)	11/134 (8%) [a]	15/134 (11%)
Age						
< 80 years	53/222 (24%)	19/222 (9%)	27/222 (12%)	81/207 (39%)	26/207 (13%)	25/207 (12%)
≥ 80 years	75/213 (35%) [a]	39/213 (18%) [a]	41/213 (19%)	13/21 (62%)	2/21 (10%)	1/21 (5%)
ASA						
I/II	54/209 (26%)	21/209 (10%)	29/209 (14%)	56/139 (40%)	17/139 (12%)	14/139 (10%)
III/IV	73/208 (35%) [a]	34/208 (16%)	38/208 (18%)	32/75 (43%)	10/75 (13%)	12/75 (16%)
Approach						
Non-posterolateral	31/165 (19%)	25/165 (15%)	30/165 (18%)	24/74 (32%)	9/74 (12%)	11/74 (15%)
Posterolateral	96/262 (37%) [a]	32/292 (12%)	38/262 (15%)	70/152 (46%)	18/152 (12%)	15/152 (10%)
Fixation						
Cemented	81/243 (33%)	5/243 (2%)	52/243 (21%)	42/82 (51%)	5/82 (6%)	11/82 (13%)
Uncemented	46/183 (25%)	52/183 (28%)*	16/183 (9%)*	51/142 (36%) [a]	22/142 (15%) [a]	14/142 (10%)

[a] P ≤ 0.05.

 Acta Orthopaedica 2018; 89 (DOI: 10.1080/17453674.2018.1499069)

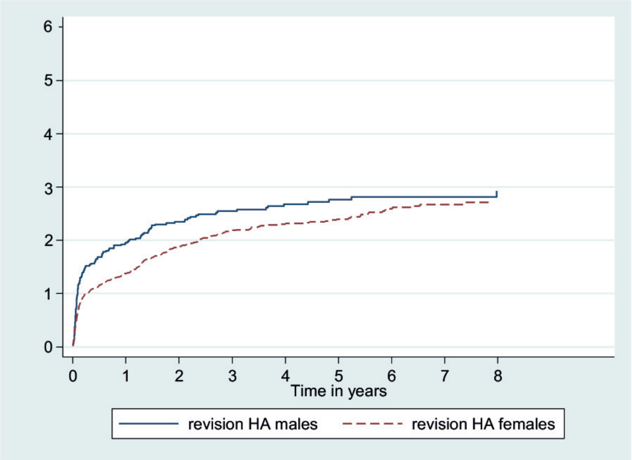

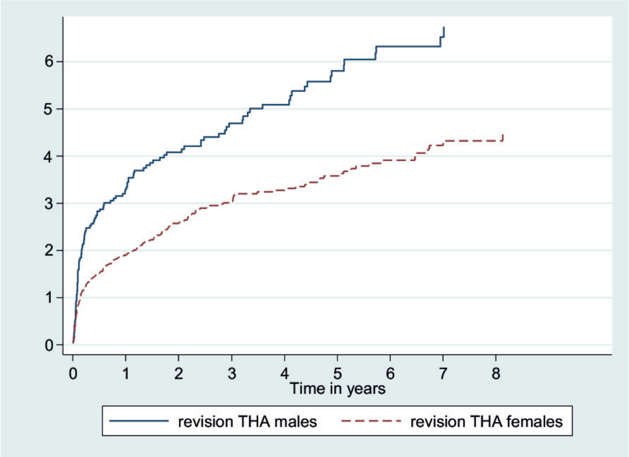

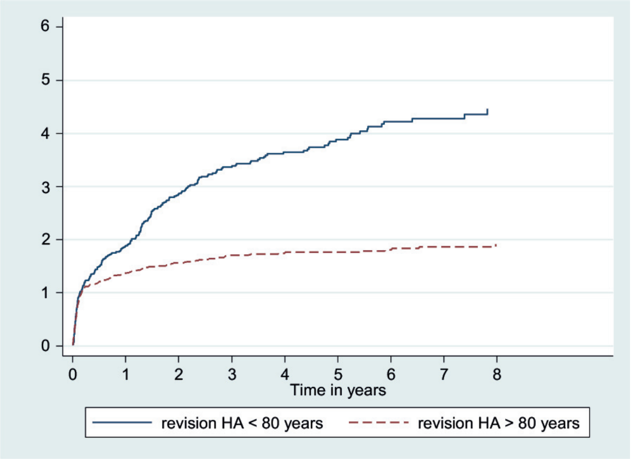

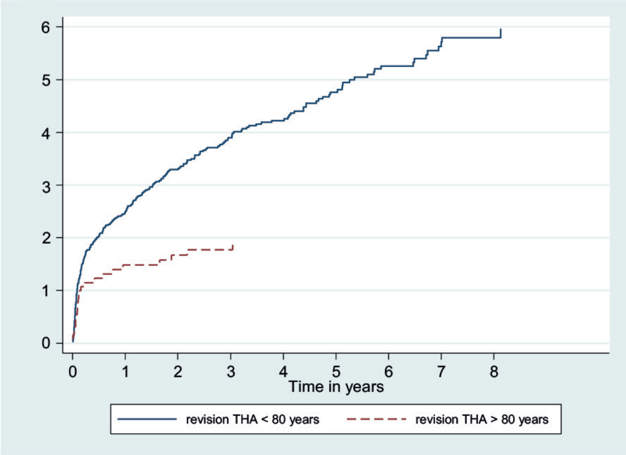

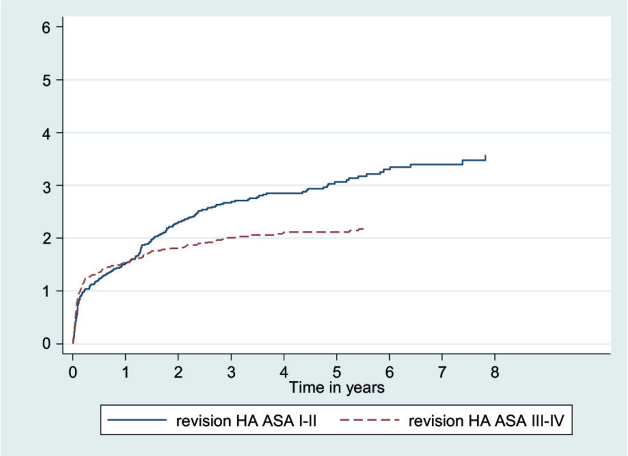

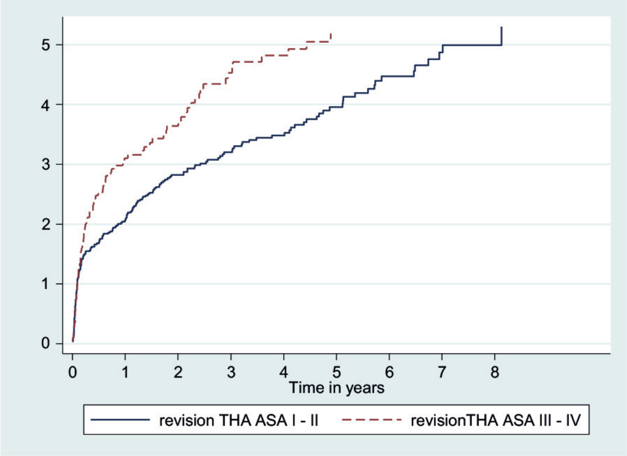

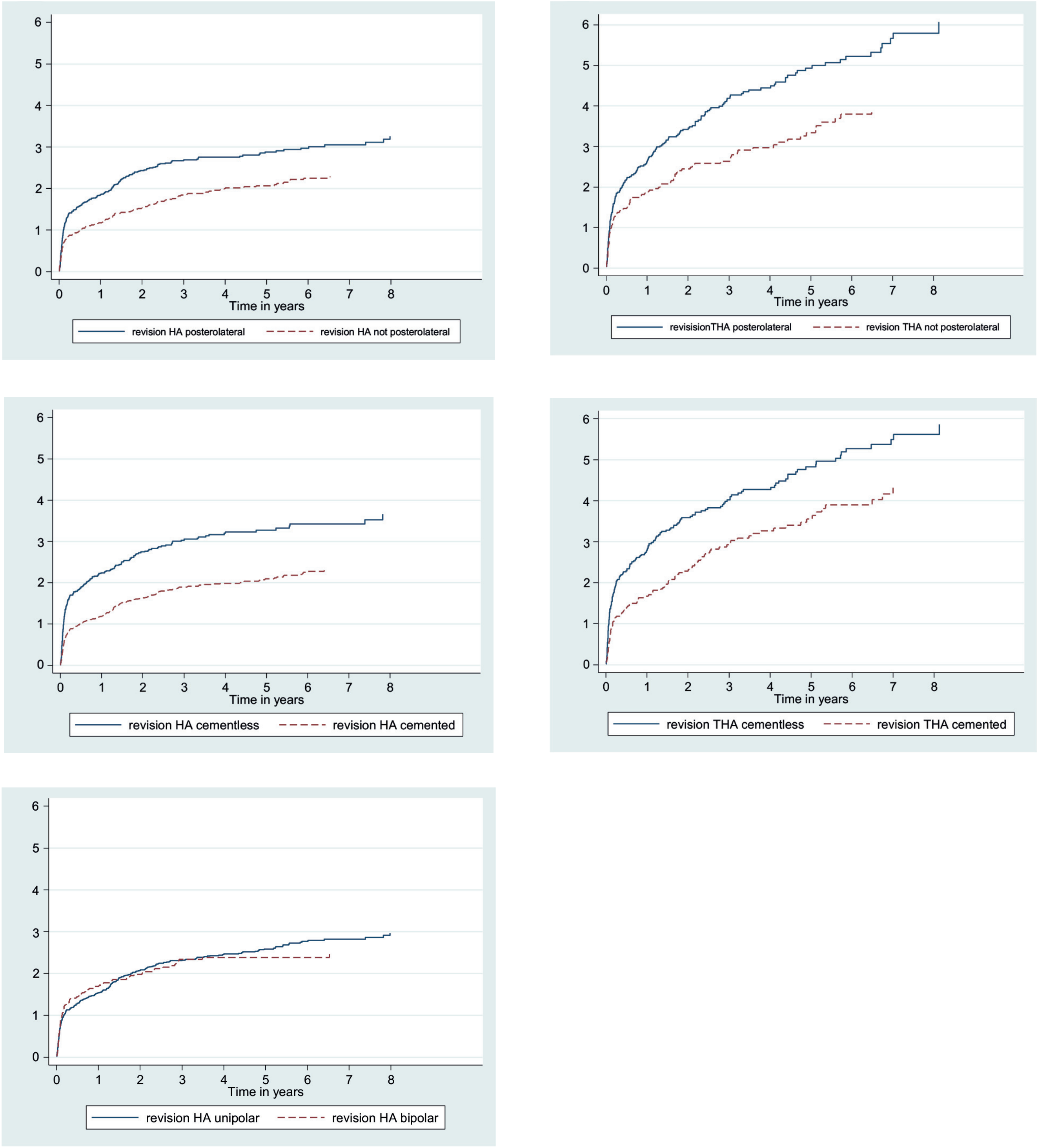

Figure 2. Cause-specific hazard for revision for patients with a hip fracture treated with a total hip arthroplasty (THA) or a hemiarthroplasty (HA).
